# Effects of screening and brief intervention training on resident and faculty alcohol intervention behaviours: a pre- post-intervention assessment

**DOI:** 10.1186/1471-2296-6-46

**Published:** 2005-11-04

**Authors:** J Paul Seale, Sylvia Shellenberger, John M Boltri, IS Okosun, Barbara Barton

**Affiliations:** 1 Department of Family Medicine, Mercer University School of Medicine and Medical Center of Central Georgia, 3780 Eisenhower Parkway, Macon GA 31210, USA; 2 Institute of Public Health, Georgia State University, One Park Place South, Sixth Floor, Suite 660, Atlanta, GA 30302, USA

## Abstract

**Background:**

Many hazardous and harmful drinkers do not receive clinician advice to reduce their drinking. Previous studies suggest under-detection and clinician reluctance to intervene despite awareness of problem drinking (PD). The Healthy Habits Project previously reported chart review data documenting increased screening and intervention with hazardous and harmful drinkers after training clinicians and implementing routine screening. This report describes the impact of the Healthy Habits training program on clinicians' rates of identification of PD, level of certainty in identifying PD and the proportion of patients given advice to reduce alcohol use, based on self-report data using clinician exit questionnaires.

**Methods:**

28 residents and 10 faculty in a family medicine residency clinic completed four cycles of clinician exit interview questionnaires before and after screening and intervention training. Rates of identifying PD, level of diagnostic certainty, and frequency of advice to reduce drinking were compared across intervention status (pre vs. post). Findings were compared with rates of PD and advice to reduce drinking documented on chart review.

**Results:**

1,052 clinician exit questionnaires were collected. There were no significant differences in rates of PD identified before and after intervention (9.8% vs. 7.4%, p = .308). Faculty demonstrated greater certainty in PD diagnoses than residents (p = .028) and gave more advice to reduce drinking (p = .042) throughout the program. Faculty and residents reported higher levels of diagnostic certainty after training (p = .039 and .030, respectively). After training, residents showed greater increases than faculty in the percentage of patients given advice to reduce drinking (p = .038), and patients felt to be problem drinkers were significantly more likely to receive advice to reduce drinking by all clinicians (50% vs. 75%, p = .047). The number of patients receiving advice to reduce drinking after program implementation exceeded the number of patients felt to be problem drinkers. Recognition rates of PD were four to eight times higher than rates documented on chart review (p = .028).

**Conclusion:**

This program resulted in greater clinician certainty in diagnosing PD and increases in the number of patients with PD who received advice to reduce drinking. Future programs should include booster training sessions and emphasize documentation of PD and brief intervention.

## Background

Patients with hazardous and harmful drinking patterns are commonly encountered by primary care clinicians worldwide. Studies from the U.S., Europe and Australia indicate that 10–40% of patients seen in primary care settings engage in hazardous or harmful drinking [1-5]. This group of patients, sometimes referred to as "risky drinkers," includes patients who meet diagnostic criteria for alcohol abuse and alcohol dependence, as well as patients who exceed recommended "safe drinking guidelines" of the National Institute for Alcohol Abuse and Alcoholism and are at increased risk for alcohol-related problems [2]. Numerous randomized controlled trials have demonstrated that screening and brief intervention (SBI) are effective in reducing alcohol consumption among such drinkers [6-8], yet SBI still remains underutilized in primary care practices. Several older studies, many of them based on chart review data, suggested that problem drinking (PD) was largely undetected and untreated in primary care [9-12]. Two recent studies using other measurement techniques (clinician exit questionnaires and direct observation) have indicated that discussions of alcohol use by clinicians and patients in primary care are more frequent than previously thought, occurring in 9–10% of primary care encounters [13,14]. Nonetheless, studies conducted in the U.S., Australia, the United Kingdom and Finland indicate that clinicians frequently fail to screen for PD, and fail to address PD in at least one-third to one-half of cases, even when the diagnosis is known [13,15-20]. Indeed, 72% of U.S. primary physicians surveyed in 1999 reported that they preferred not to counsel early problem drinkers themselves but rather to refer them to a nurse trained in behavioural interventions [21]. Residents are less likely to perform brief interventions than faculty physicians [22], and only 13–20% of problem drinkers report receiving advice to reduce drinking, a key element of most effective SBI programs [17,22,23]. While studies have demonstrated that providing experiential training can increase primary care clinicians' rates of providing brief advice to problem drinkers [24-27], studies of the effect of resident training have yielded mixed results [28,29]. We previously reported initial findings from the Healthy Habits Project, a training program designed to increase SBI rates in a family medicine residency program using a combination of clinician training and clinic systems intervention. Based primarily on chart review, findings indicated that following program implementation, alcohol interventions increased from 12.5% to 47.7% of patients who screened positive for hazardous and harmful drinking, and that clinicians who were prompted with positive screening results gave advice to reduce drinking to 72% of patients [30]. This report provides further information, obtained using clinician exit questionnaires, regarding the impact of this SBI training program on resident and faculty physician alcohol intervention attitudes and behaviours. We hypothesized that the SBI training program would result in the following changes for both resident and faculty clinicians: (1) greater recognition of PD, (2) increased certainty in identifying PD, and (3) increased advice to reduce drinking.

## Methods

The Healthy Habits Project utilized a combination of clinician training and a clinic-wide systems intervention program to increase alcohol screening and brief intervention in a family practice residency clinic in the southeastern U.S. Details of the program's systems interventions and training procedures have been previously described [30]. Briefly, the clinic is staffed by residents (28 physicians completing three years of post medical school training) and by faculty (8 family physicians and 2 physician assistants). All of the clinic's clinicians (residents and faculty) participated and gave written consent, and efforts were made to screen all adult patients during a 12-month period. The study was approved by the Institutional Review Board of the Medical Center of Central Georgia. Alcohol screening and intervention procedures were modeled after the University of Connecticut's Cutting Back Screening and Brief Intervention Program [26,31]. A clinic team implemented alcohol screening using the three-question AUDIT-C, a validated screening instrument for hazardous and harmful drinking [32-34], embedded in a health questionnaire distributed by registration clerks. After scoring the AUDIT-C, nurses asked screen-positive patients to complete the ten-question Alcohol Use Disorders Identification Test (AUDIT) [35]. All clinicians underwent 3 hours of training in which they were instructed to score the 10-question AUDIT and conduct brochure-guided brief interventions with all screen-positive patients. A key component of the intervention was negotiating a clinician-patient contract to reduce alcohol consumption during the ensuing 30 days. Training included a lecture, demonstration interviews, and role-playing exercises. Clinicians were asked to reschedule a follow-up visit within 30 days. Regular feedback sessions with clinic staff, nurses, and clinicians were implemented to encourage compliance with protocols. Periodic program evaluation by the project's implementation committee resulted in minor modifications of protocols which created three separate implementation phases: A (Months 1–3), B (Months 4–6), and C (Months 7–12). During Phase B, in an attempt to boost overall patient screening rates, monthly feedback sessions were scheduled with clinic staff and nurses. During Phase C, as a further attempt to increase screening rates, alcohol screening questions were integrated into the clinic's mandatory annual clinical information update.

### Assessments

#### Clinician exit questionnaires (CEQ's)

A pre-post research design was used to measure the project's impact on clinicians' level of certainty in identifying problem drinking and their self-report of advice given to patients to reduce their drinking. Four sets of CEQ's were collected (longitudinally during the baseline month and Implementation Phase A, then for 1–2 weeks at the end of Phases B and C). Clinicians were asked to complete a three-question instrument (see Appendix 1) in which they reported whether they thought the patient they had just seen had a drinking problem, their degree of certainty of this diagnosis on a five-point Likert-type scale, and whether they had suggested that the patient stop drinking or cut back. All CEQ's were completed at the conclusion of individual patient encounters. The first, second, and fourth collections were performed by a research assistant, who approached individual clinicians in the hall immediately following two patient visits during each half-day clinic session. During the third collection period (Phase B), exit questionnaires were attached to each patient's routing form, and clinicians were requested to complete them on each patient seen.

#### Chart reviews

During two one-month periods (at baseline and during the project's final month), chart reviews of every fourth adult patient seen were conducted by one of two investigators (JPS, BB), who reviewed clinic notes from the patient's database, problem list, index visit, and all office visits during the previous year. Patients were considered "diagnosed" if alcohol abuse, alcohol dependence, heavy drinking, or similar terms were listed in the assessment area of a clinic note or the patient's problem list. Documentation of advice to quit drinking, cut back, or attend formal alcohol treatment was considered "intervention."

### Statistical analysis

Statistical programs available in SPSS for Windows were used for analysis [36]. Pre- and post-assessments of the clinician's diagnostic impression, degree of certainty, and advice to decrease drinking were compared. Data were analyzed separately for faculty and residents, and then for all clinicians. Data from the three implementation phases of the study were analyzed separately and as aggregate data. Additional analyses were performed after removing CEQ's completed by two of the study's co-investigators (JPS, JB) to look for possible bias. All data were analyzed using Pearson's Chi-squared test. For the tables with expected cell frequencies less than five, Fisher's exact test was employed. Chi-square test for trend was used to determine diagnostic changes over time for faculty and residents. Analysis of variance (ANOVA) was used to compare differences between faculty and residents across baseline and study phases for the study's three primary outcome measures – mean number of subjects recognized with problem drinking, level of certainty in recognition, and number of patients given advice to reduce drinking. ANOVA was also used to assess possible impact of clinician gender and age on these three outcome measures. Test of linear trends was used to assess whether clinical diagnoses of problem drinking increased or decreased over time for patients who were evaluated by faculty, residents and all clinicians. The customary p-values of < 0.05 were used to indicate statistical significance.

## Results

Thirty-eight clinicians completed exit questionnaires from a total of 1,052 patient encounters (164 at baseline, 888 during the implementation period). Residents were significantly younger than faculty clinicians (mean age 34 vs. 44 years, p < .0001; see Table 1). Although a higher percentage of residents were female, when compared to faculty, (64% vs. 30%), differences were not statistically significant (p = .078). During the third data collection period, in which exit questionnaires were self-administered, questionnaires were completed on 44% of adult patients seen during a one-week period. Chart reviews were conducted on 178 charts from the one-month baseline period and 200 charts from Month 12 of implementation.

**Table 1:** Baseline comparability: demographics of residents and faculty

**Variable**	**Faculty (n = 10)**	**Residents (n = 28)**	**Total Participants (n = 38)**
**Age, years mean (SD)**	44.2 (SD 6.4)	34.0 (SD 6.6)	36.7 (SD 7.9)
**% Female**	30.0	64.3	55.3

### Recognition of problem drinking

Overall, there was no statistically significant difference in mean number of subjects recognized as problem drinkers by faculty and residents across baseline and study phases. Clinicians reported problem drinking in 9.8% (16/164) of patients at baseline and 7.4% (66/888) of patients during the project's three implementation phases (p = .308). These rates are similar to the rates of risky drinking obtained by questionnaire screening using the AUDIT-C (8.6% during the baseline period and 8.0% during the implementation period [30]). Faculty impressions of problem drinking remained relatively stable throughout the project, while residents' diagnostic impressions showed a trend toward decline over time (see Table 2; p = .052). Despite equivalent percentages of patients who screened positive for hazardous drinking using the AUDIT-C at baseline (8.6%) and during the final phase of the study (8.8%), recognition rates for PD measured by CEQ were significantly higher than rates documented in patient charts during both periods (baseline: 9.8% vs. 1.2%, p = .035, and final study phase: 6.1% vs. 1.5%, p = .008).

**Table 2:** Changes in numbers and percent of patients diagnosed as problem drinkers across study phases

	**Diagnoses by faculty ** **N (%)**	**Diagnoses by residents ** **N (%)**	**Diagnoses by all clinicians ** **N (%)**
**Study Phase**			
**Baseline**	77 (9.1)	86 (10.5)	164 (9.8)
**Phase A**	222 (9.5)	279 (7.5)	501 (8.4)
**Phase B**	141 (5.7)	90 (6.7)	256 (6.3)
**Phase C**	78 (10.2)	43 (0)	131 (6.1)
**p-value**	.782	.052	.148

p-value is for linear trends

### Certainty in identifying problem drinking

Clinicians' mean level of certainty regarding the presence or absence of hazardous or harmful drinking among all patients was high both before (4.23, +/- 0.98) and after SBI implementation (4.21, +/- 0.97). ANOVA showed no differences between certainty levels for faculty and residents across all patient encounters (p = .446). Differences were observed, however, when analyses were limited to patients felt to have a drinking problem. Faculty level of certainty in patients with PD was greater than residents' level of certainty both before SBI program implementation (4.14 vs. 3.56) and after implementation (4.38 vs. 3.96), p = .028. After program implementation, levels of certainty for patients with PD increased for both faculty (4.38 vs. 4.14, p = .039) and residents (3.96 vs. 3.56, p = .030).

### Advice to reduce drinking

**Figure 1:**
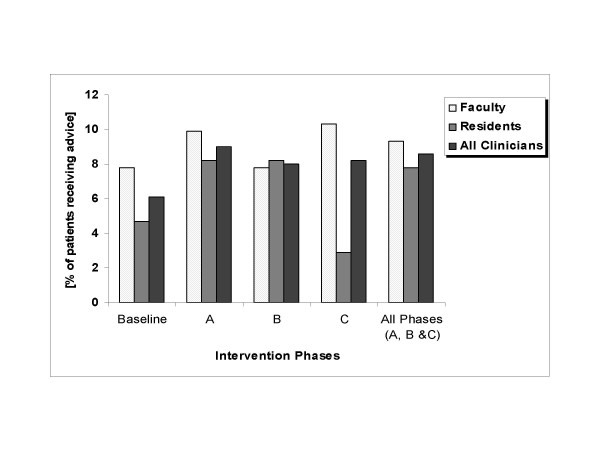
Changes in clinician advice to reduce drinking (all patients).

Overall, comparative analysis of mean number of patients given advice to reduce drinking by faculty and residents across baseline and study phases did not demonstrate statistically significant changes. CEQ responses indicated that clinicians gave brief advice to 6.1% (10/164) of all patients seen at baseline and 8.6% (75/874) of patients seen during the project's implementation phase (p = .287); see Figure 1. Analyses revealed no impact of gender or clinician age on clinician advice rates. Intervention rates were highest during Phase A, the first three months following training. Rates decreased modestly for all providers during Phase B, then in Phase C showed declines for residents and increases for faculty. Comparisons of the mean number of all subjects receiving advice to reduce drinking by faculty vs. residents across study phases found that faculty were significantly more likely to advise patients to reduce drinking (p = .042). When baseline brief advice rates are compared with those during the combined intervention periods, residents showed greater increases in brief advice rates (from 4.7 % to 7.8%) than faculty (from 7.8 % to 9.3%); p = .041. Intervention rates increased among faculty who were co-investigators in the study (11.5% vs. 16.7%, p = .049) but showed no significant change among faculty who were not (9.8% vs. 7.0%, p = .421). When the analysis was limited to patients felt by clinicians to be problem drinkers, advice to reduce drinking increased from 50% (8/16) of problem drinkers during the baseline phase to 75% (49/65) during the three implementation phases (p = .038). While the overall number of patients receiving advice to reduce drinking was less than the number of patients thought to be problem drinkers during the baseline period (10/16, or 62%), the number of patients receiving advice to reduce drinking during the implementation phase actually exceeded the number of patients thought to be problem drinkers (75/66, or 114%).

### Effect of clinician age and gender

Finally, ANOVA showed no statistically significant effect of age or gender of the faculty and residents on the study's three primary outcome measures – mean number of subjects recognized with problem drinking, level of certainty in recognition, and number of patients given advice to reduce drinking.

## Discussion

### Clinician attitudes and behaviours related to brief intervention

To our knowledge, this study is the third residency-based SBI training program to demonstrate positive changes in clinician attitudes and behaviours related to alcohol intervention. Researchers at the University of Massachusetts previously reported increases in readiness to intervene and in actual performance of brief interventions performed by residents and faculty physicians in a similar program which also provided clinician training, routine screening, and clinician prompting with screen-positive patients [24,37]. Wilk and Jensen [28] reported increases in brief interventions by residents in interviews with unannounced standardized patients following brief intervention training. In our study, both faculty and residents showed significant increases in their certainty in diagnosing PD after training. Residents showed greater increases in diagnostic certainty and intervention rates than faculty, an important finding in light of evidence from our study and others [22] that residents are less likely than faculty to perform brief interventions. Because previous studies have demonstrated that not all alcohol-related discussions include advice to reduce drinking, a key element of SBI cited by the US Preventive Services Task Force [38], our evaluation was focused on the percentage of patients who actually received advice to reduce drinking. During the project's intervention phase, clinicians reported a modest increase in providing advice to reduce drinking (from 6.8% to 8.6%). While this increase did not reach statistical significance (p = .287), a significant increase was seen in the percent of perceived problem drinkers receiving such advice (50% to 75%, p = .047). These findings, although based on small numbers of encounters with problem drinkers, are consistent with previous studies indicating that clinicians who have received SBI training are more confident in their ability to conduct brief interventions and more likely to intervene with problem drinkers [26,39,40]. In contrast to some earlier studies which found younger clinicians to be more willing to intervene than older clinicians [39,41,42], this study found no impact of age or gender on confidence in diagnoses of problem drinking or advice given to reduce drinking. Reasons for this finding are unclear, but could be related to the relatively young age of the overall group (mean age 37, with only two clinicians over age 50) or to the fact that clinicians of all ages received intensive training, which has been shown to correlate with greater clinician confidence and performance levels [40,42,43]. Further research is needed to determine which of the training program's multiple components – experiential training, implementation of routine alcohol screening performed by nurses, prompting clinicians with positive screening results and assessment data, or compliance feedback regarding intervention rates – were most critical in achieving increased intervention rates.

Overall, our study indicated that faculty prescribed reduction in drinking to more patients than residents did, but that resident intervention rates showed greater increases after training than faculty rates. Resident interventions did, however, show a non-significant trend toward decline toward the end of the one-year study. While this could represent a loss of training effect over time, evaluation results also may have been confounded by conducting the final exit interview evaluation in July, when skilled third-year residents had just graduated and newly-promoted residents were struggling to manage increased patient volumes. Interestingly, intervention prompt forms reviewed for the previous report on this project [30] indicate that there was no decline in resident interventions when prompted with positive screening results during this period: brief interventions were performed in 75% (6/8) of cases. Regardless of the reason for the decline, findings suggest that reinforcement methods such as booster sessions are needed to maintain behaviours taught in the initial training sessions.

One of this study's most encouraging findings is the fact that the number of patients receiving advice to reduce drinking after SBI training actually exceeded the number of patients felt to be problem drinkers. This finding suggests that the SBI program was successful in legitimizing and normalizing conversations about alcohol, such that clinicians felt more at ease in addressing alcohol use in a variety of clinical scenarios, and indicates that at-risk drinkers as well as problem drinkers received brief advice to reduce their drinking.

### Recognition of problem drinking

In contrast to our expectation, clinician exit questionnaires did not reflect increased recognition of problem drinking after program implementation. While this could be due to the relatively high levels of clinician recognition at baseline, it could also reflect decreased clinician vigilance once routine screening protocols were in place. This finding, if confirmed in other studies, has implications for future SBI training programs, especially in light of the fact that most "routine" screening systems are not effective in detecting all risky drinkers. Clinician training should include reminders that significant numbers of risky drinkers may remain unscreened or escape detection via questionnaire screening, whose sensitivity rarely exceeds 85%, and clinicians must remain alert to clinical clues related to hazardous or problem drinking.

### Documentation of problem drinking

One striking finding of this study is the marked difference between the perceived level of problem drinking (7.4–9.8% of patients) and the documentation of such diagnoses in the medical record (1.2–1.5%), suggesting that clinicians document perceived problem drinking in less than 25% of cases. Significant underdocumentation of alcohol disorders has been noted in several other previous studies [15,44,45], pointing out an important methodological flaw in many previous studies which have presumed that problem drinkers are "undiagnosed" based on chart review data [9-12]. This finding suggests that future studies assessing clinician recognition and intervention rates should utilize more sensitive measures such as clinician or patient exit interviews or direct observation, and that future training efforts should address documentation of alcohol disorders in the medical record.

### Limitations of this pilot study

It is important to take into account possible methodological limitations of this pilot study. First, the sample size was not large, particularly during the baseline assessment period. However, the numbers in each group were sufficient to detect between-group differences of at least 10% or greater in recognition of problem drinking and at least 10% or greater in advice to reduce drinking. Secondly, a change in methodology during the study's second implementation phase could have confounded study results. During this period, exit questionnaires were attached to each patient's routing form, and clinicians were requested to complete them on each patient seen. The return rate was low (44%), and selection bias may have occurred, perhaps in favour of patients whom clinicians identified as problem drinkers or in favour of patients who received an intervention. Nonetheless, both clinicians' recognition (6.7% of patients) and intervention rates (8.0%) during this phase are within the range of the other phases of the study and do not suggest selection bias. Thirdly, this study lacked a criterion diagnosis for confirming problem drinking in patients considered by clinicians to be problem drinkers. While some patients may have been incorrectly diagnosed, rates of problem drinking recognition by clinicians throughout this study are similar to this study's previously-published problem drinking estimates obtained by AUDIT-C questionnaire screening, and only slightly lower than the estimated U.S. problem drinking prevalence of 11% in primary care [1]. Future studies comparing clinician's impressions with the results of standardized diagnostic interviews for problem drinking could help to clarify this issue.

## Conclusion

This SBI training program resulted in greater clinician certainty in diagnosing PD and modest but significant increases in the number of patients with PD who received advice to reduce their drinking. The program shows promise for helping translate SBI findings into residency and clinical practice. Trends toward lower rates of identification of PD and intervention by residents during the program's later phases suggest a need for booster training sessions. Increased emphasis on documentation of problem drinking and brief intervention is also needed.

## Appendix 1: exit questionnaire for clinicians

1). Do you think this patient has a drinking problem? Yes__ No

2). What is your degree of certainty?

Uncertain 1     2     3     4     5     Certain

3. Did you talk with this patient today about cutting back or quitting?

No___ Yes____

## Competing interests

The author(s) declare that they have no competing interests.

## Authors' contributions

JPS conceived of and designed the study, supervised acquisition of the data, participated in interpretation of data and drafted the manuscript. SS assisted in designing and implementing the study, interpreting the data, and editing the manuscript. JMB assisted in designing and implementing the study, interpreting the data and editing of the manuscript. ISO performed the statistical analysis and participated in drafting and editing the manuscript. BB participated in data collection, study design and implementation, and data analysis.

## Pre-publication history

The pre-publication history for this paper can be accessed here:


